# Expression of phosphohistone H3 and X-linked inhibitors of apoptosis-related proteins in mucoepidermoid carcinoma and adenoid cystic carcinoma of salivary glands

**DOI:** 10.1038/s41598-025-21234-9

**Published:** 2025-11-18

**Authors:** Aya Alsaiedy, Heba EL-Hendawy, Rehab Eldesoky, Nora El-Tantawy, Azza El-Sissi

**Affiliations:** 1 Faculty of Dentistry, October University for Modern Sciences and Arts (MSA), Giza, Egypt; 2 Department of Oral Pathology, Faculty of Dentistry, Mansoura University, Mansoura, Egypt; 3 Department of Anatomical Pathology, Faculty of Medicine, Mansoura University, Mansoura, Egypt; 4 Department of Public Health, Faculty of Applied Medical Sciences, Al-Baha University, Al-Baha, Saudi Arabia; 5 Department of Medical Parasitology, Faculty of Medicine, Mansoura University, Mansoura, Egypt

**Keywords:** Salivary gland, Tumors, XIAP, PHH3, Immunohistochemistry, Prognosis, Cancer, Cell biology, Molecular biology, Molecular medicine

## Abstract

The two most prevalent cancers of the salivary glands are adenoid cystic carcinoma (AdCC) and mucoepidermoid carcinoma (MEC). They show great variation in clinical presentation and histopathological features, which can influence patient prognosis. Phosphohistone H3 (PHH3) is an essential histone protein involved in chromatin structure and cell division. It has proven valuable in diagnosing and grading several tumor types. X-linked inhibitor of apoptosis protein (XIAP) prevents apoptosis. Both PHH3 and XIAP could shed light on the biological behavior prognosis of MECs and AdCCs. The purpose of this research was to assess the expression of PHH3 and XIAP in different histological types of MEC and AdCC and examine their relationships with the clinicopathological characteristics of patients. This study analyzed 30 archived paraffin-embedded tissue blocks, including 16 samples from MEC patients and 14 from AdCC patients. Immunohistochemical staining was performed to assess PHH3 and XIAP expression. The results were evaluated via a semiquantitative scoring system and statistically correlated with clinicopathological characteristics. All patients had positive immunohistochemical expression of both PHH3 and XIAP. Higher levels of these markers were directly associated with higher histological grades in both MECs and AdCCs. PHH3 and XIAP expression strongly correlated with each other. Immunoreactivity for both markers was significantly elevated in tumors with higher-grade histopathological features. The histological grade of MEC and AdCC might be affected by the expression of PHH3 and XIAP biomarkers. However, further studies with larger sample sizes are needed to confirm the possible prognostic role of PHH3 and XIAP in the diagnosis of MEC and AdCC.

## Background

Malignant salivary gland tumors (MSGTs), both major and minor, arise in the salivary glands^1^. MSGTs comprise multiple histologic entities with diverse clinical behaviors. They include more than 20 histotypes, of which adenoid cystic carcinoma (AdCC) and mucoepidermoid carcinoma (MEC) are the most prevalent primary salivary gland malignancies^2^. To confirm the histopathological diagnosis of salivary gland tumors in clinical practice, the growth pattern of the tumor borders, histological architecture, cellular structure and differentiation, and tumor stroma components are carefully assessed in addition to the clinical data.

However, new tools for immunohistochemical (IHC) diagnosis and prognostication, such as cell proliferation markers, antiapoptotic proteins, myoepithelial antigens, matrix metalloproteinases, steroid receptors, growth factors, and their receptors, have been combined with recent advances in histopathological classification. These findings have increased the probability of more specific options for treating various types of salivary gland cancers^3^.

Mucoepidermoid carcinoma (MEC) is the most common of all MSGTs and accounts for nearly 10% of any type of tumor of the salivary glands^3^. Several schemes for grading MEC are found in the literature, each having distinct advantages and disadvantages. Based on histological findings, MEC is categorized as low (well differentiated), intermediate, or high (poorly differentiated) according to the most widely used grading system^4^.

Despite the strong correlation between tumor grade and clinical stage, low-grade tumors are associated with metastases and tumor-related death^5^. Furthermore, because there is debate about the best grading criteria, studies are also widely prone to misclassification artifacts in the high-grade category.

Adenoid cystic carcinoma (AdCC) is the most prevalent malignant neoplasm of the minor salivary glands and the second most common malignant salivary gland tumor^6^. AdCC is characterized by a slow, indolent growth pattern, early perineural invasion, extensive local infiltration, frequent distant metastases, and the potential for local recurrence^7^. According to the solid components of the tumor, AdCC has historically been classified into three histological groups: cribriform, tubular, and solid. Unfortunately, all adenoid cystic carcinomas have a longer course and a poor prognosis, independent of grade or pattern^8^. Consequently, an accurate diagnosis of AdCC depends on a thorough evaluation of its biological activity^9^. For a more accurate assessment and, as a result, improved management techniques, investigations aimed at providing further suggestions and clues about the identification of proteins that regulate biological processes such as mitosis and apoptosis are necessary^3^.

The phosphorylation of serine-10 and serine-28 residues in histone H3, a nuclear core histone protein of DNA chromatin, is essential for nuclear cycle progression and chromosomal condensation during mitosis and meiosis. Phosphorylation occurs within late G2 to early prophase after late anaphase and early telophase, whereas dephosphorylation occurs gradually. As a result, histone H3 is always highly phosphorylated and positive for phosphohistone H3 (PHH3) during metaphase, but PHH3 is either not expressed at all or is minimally expressed during interphase^10^. According to previous studies, the PHH3 index increases as the grade of malignancies, such as meningioma, ovarian cancer, breast cancer, and melanoma, increases. Consequently, PHH3 staining aids in tumor grading by increasing the mitotic count in addition to having prognostic significance^11^. PHH3 is an independent prognostic factor for meningioma^12^, cutaneous melanoma^13^, and gastric cancer^14^. X-linked inhibitor of apoptosis protein is the most effectively characterized member of the inhibitor of apoptosis (IAP) family and is considered an effective inhibitor of the caspase/apoptosis pathway. High expression of this protein corresponds to poor prognosis in many cancer tissues, including prostate carcinomas^15^, acute and chronic leukemia^16^, and other types of cancers. XIAP is also closely related to the progression and aggression of malignant tumors and contributes to chemotherapy resistance^17^.

Phosphohistone H3 (PHH3) plays a pivotal role in chromatin condensation during mitosis. Its expression level reflects the proportion of cells undergoing active cell division and has been widely validated as a sensitive marker of mitotic activity in various malignancies^10–14^. In contrast, XIAP is a potent anti-apoptotic molecule belonging to the inhibitor of apoptosis protein (IAP) family. XIAP directly binds to and inhibits caspases-3, -7, and -9, thereby blocking the execution phase of apoptosis and promoting tumour cell survival^15–17^. The combined overexpression of PHH3 and XIAP may therefore represent a dual biological mechanism in tumour progression: PHH3 indicating heightened proliferative drive, and XIAP preventing programmed cell death. In salivary gland malignancies such as MEC and AdCC, this proliferative–antiapoptotic synergy could contribute to more aggressive tumour phenotypes, higher histologic grades, and poorer prognoses. Considering these findings, the present study investigated the variation in mitotic and antiapoptotic activities reflected in the immunohistochemical (IHC) expression of the PHH3 and XIAP proteins, respectively, concerning the histopathology of both MECs and AdCCs of the salivary glands.

## Methods

### Study design and samples

This retrospective study was carried out from June 2023 to June 2024. The study was conducted after the approval of the Institutional Review Board of the Faculty of Dentistry, Mansoura University, Egypt (A09060421OP) was obtained. Informed consent was obtained from all individuals whose tissue blocks were included in the study. Each experiment was carried out in compliance with the relevant rules and guidelines. The study included thirty formalin-fixed paraffin blocks of MECs and AdCCs from the salivary glands. They were obtained from the archives of the Oncology Unit of the Oncology Center, Faculty of Medicine, Mansoura University, Egypt. The study sample was selected to comprise two groups: the first group included 16 blocks of MEC representing the three histologic grades according to the criteria of the modified Healey classification^18^. MEC cases were graded according to the modified Healey classification, which assigns points based on specific histopathological parameters, including the proportion of intracystic component, degree of nuclear atypia, number of mitoses per 10 high-power fields, presence of necrosis, and perineural invasion. Each feature is scored, and the sum of scores determines the final grade: low grade (0–4 points), intermediate grade (5–6 points), and high grade (7–14 points).

In this study, three, three, and ten blocks for low, intermediate and high grades of MEC; the second group included 14 blocks for AdCC representing the three histologic grades according to the criteria of Perzin et al.^19^ and Szanto et al.^20^ as follows: 1 block for low grade (characterized by the predominant tubular pattern with no solid component), 5 blocks for intermediate grade (revealed a predominant cribriform pattern with a solid component < 30%) and 8 blocks for high grade (presented a solid component in > 30% of the examined carcinoma). The recorded clinical and demographic data were obtained from the registration files of the Mansoura Oncology Center at Mansoura University, Egypt. Each of the 30 paraffin blocks was used to prepare four-micron-thick three-tissue sections to perform routine hematoxylin and eosin (H&E) staining of one section to review and confirm the diagnosis of the studied cases according to Bancroft and Gamble^21^. Second, the second and third sections were subjected to immunohistochemical staining.

### Immunohistochemical staining

One of the two sections was used for immunostaining for the XIAP marker, and the other one was used for immunostaining for PHH3. To promote tissue adherence to the slide surface, sections for IH staining were placed on electrically charged, positively charged slides. Immunostaining was performed via the avidin–biotin complex (ABC) technique^22^ via the universal streptavidin–biotin kit (ultravision detection system) as directed by the manufacturer’s guidelines. Phosphohistone H3 (PHH3) reagent (phospho-histone H3-S10 rabbit mAb, ABcLonal, Catalog No. AP0002, USA, dilution 1:100) was provided in a vial of 20 μl of concentrated rabbit polyclonal antibodies. The suggested and used dilution was 1:100. X-linked inhibitor of apoptosis protein (XIAP) reagent ([KO Validated] XIAP Rabbit pAb, ABcLonal, Catalog No. A6869, USA) was provided in a vial containing 20 μl of concentrated rabbit polyclonal antibodies. The suggested and used dilution was 1:100.

In brief, the slides were first deparaffinized in xylene for 15 min, rehydrated with decreasing alcohol concentrations, and then rinsed with water. The tissue sections were treated with 0.5% hydrogen peroxide in methanol for 30 min to block endogenous peroxidase, followed by a 5-min PBS rinse. Antigen retrieval was conducted by boiling the slides in sodium citrate buffer at 94 °C for 20 min, cooling them to room temperature, and rinsing them with distilled water. The slides were preheated to 37 °C, incubated with protease XIV solution to eliminate proteolytic enzyme activity, and then blocked with 4.0% mouse serum for 30 min to prevent nonspecific antibody binding. Primary antibodies (anti-XIAP and anti-PHH3) were applied at optimal dilutions and incubated overnight at 4 °C. Afterward, the samples were rinsed, and a biotinylated secondary antibody was applied for 30 min, followed by three washes with PBS. The avidin–biotin complex peroxidase solution was then utilized in accordance with the manufacturer’s instructions and incubated for 30 min, followed by rinsing. To visualize the target antigens, DAB chromogen was used, producing a brown reaction product after a 15-min incubation and three PBS washes. The sections were then mounted with DPX and counterstained with Mayer’s hematoxylin. Under the same conditions, a positive control slide was included in every batch. A portion of ovarian cancer samples was used as the PHH3 antibody-positive control, whereas a portion of kidney cancer samples was used as the XIAP-positive control. For each batch, a negative control slide was used, with plain PBS used in place of the primary antibodies.

### Localization of the IH-positive immunoreactivity

**Fig. 1:**
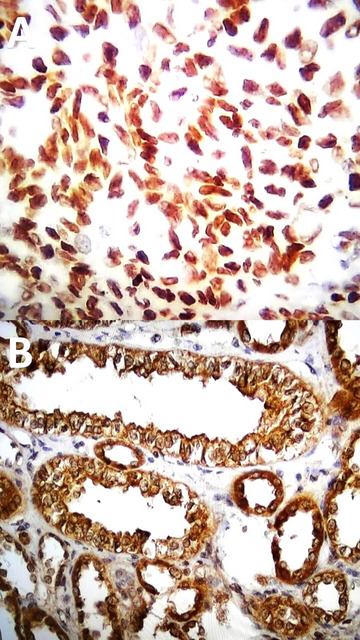
(**A**) Strong nuclear PHH3 expression in ovarian cancer tissue (Positive control, ABC-DAB, 400×); (**B**) strong nuclear and cytoplasmic XIAP expression in kidney cancer tissue (Positive control, ABC-DAB, 400×).

The IH assessment of both PHH3 and XIAP expression was conducted by scanning each slide under low magnification (×100) to identify areas containing positive immunoreactivity. The positive immunoreaction for PHH3 was predominantly encountered intranucleously (Fig. 1A), whereas XIAP was found in both the nucleus and the cytoplasm (Fig. 1B).

### Assessment and grading of the immunohistochemistry results

All the samples were examined and scored for immunoreactivity by two pathologists who were blinded to the clinicopathological data, and discrepancies were resolved by consensus. While stromal and inflammatory cells were not scored for any staining, epithelial cells were scored. The immunoreactivity of PHH3 was determined via the method of Cui et al.^23^, where the total number of tumor cells in 10 consecutive HPFs with the highest immunolabeling prevalence (hot spot) was used to grade the PHH3 MI. However, the number of counted fields in the current work was five. The following were the mitotic score thresholds for mitotic counts: 9 cells received a score of 1, 10–19 cells received a score of 2, and more than 20 cells received a score of 3.

The immunoreactivity of XIAP was determined via the method of Xu YC et al.^24^. A semiquantitative scoring system with four tiers was used to score XIAP staining since both the staining intensity and extent of positivity as follows: A semiquantitative scale was used to assess the staining intensity: 1+ for weak, 2+ for moderate, and 3+ for strong. The degree of staining was rated as follows: 0 for less than 5%, 1 for focal (6–25%), 2 for multifocal (26–50%), 3 for regional (51–75%), and 4 for diffuse (76–100%). The final immunoreactivity score was then determined by multiplying the stain intensity by the extent of the stain. Scores of 0–2 were considered negative, scores of 3–5 were considered weak, scores of 6–8 were considered moderate, and scores greater than or equal to 9 were considered strong.

### Statistical analysis

The results of the study were statistically analyzed to find any possible significant variations or correlations between the different variables. The data were statistically analyzed via Excel and the Statistical Package for the Social Sciences (SPSS) version 22 software.

Frequencies and proportions were used to represent qualitative data, whereas the mean (+/−) standard deviation (SD) was used to describe quantitative data. Pearson’s chi-square test and one-way ANOVA were used to analyze the data and compare the differences between the groups under study. The relationships among the various variables were investigated via the Pearson correlation coefficient test. A p-value of less than 0.05 was considered statistically significant.

## Results

### Clinical findings

Three males (18.75%) and thirteen females (81.25%) represented the current study’s MEC group (16 cases), which had a mean age of 61 years and ranged in age from 37 to 76 years. The majority of MEC patients (87.5%) manifested MEC in the main salivary glands. The majority of the MEC patients (81.25%) had a reported clinical stage that ranged between stages III and IV. With a mean age of 56 years and a range of 49–74 years, the AdCC group (14 cases) had a comparable number of males and females. AdCC was slightly more common (57.1%) in the major salivary glands (mainly the parotid) than in the minor salivary glands (42.8%). Small-sized tumors (T1 and T2) were reported in one patient (7%). The dominant (92.8%) presentation of AdCC was among the stage groups III and IV. A single expected case was reported as clinical stage II.

### Histopathological findings

Three cell types—mucous-secreting cells, epidermoid cells, and intermediate cells—were found in varying amounts in mucoepidermoid cancer. This group was categorized according to the modified Healey system’s criteria into three histologic grades: low, moderate, and high^25^. While low- and intermediate-grade MECs represented three cases per grade (18.8%), high-grade MECs accounted for the largest percentage of worked MEC cases (10 cases, 62.4%). Variation was observed in MEC grading based on the degree of cellular atypia, the relative proportion of constituent cells, and the number of cystic spaces.

In 14 cases (46.7%) of AdCC, the tumors presented both myoepithelial and basaloid cells arranged in cribriform, tubular, and solid patterns. In accordance with Szanto et al.^20^ and Perzin et al.^19^, tumors were graded into three histologic categories. Grade 1 (low-grade) tumors were observed in one patient (7.14%) with a predominantly tubular pattern and no solid component; these tumors had small duct-like structures lined by luminal cuboidal cells and abluminal myoepithelial cells in a hyalinized stroma. Five cases (35.7%) were grade 2 (intermediate) and featured a predominant cribriform pattern with less than 30% solid areas; some necrosis was present. Grade 3 tumors (high grade) were observed in eight patients (57%) and presented solid components exceeding 30%, with large sheets of tumor cells, minimal cystic spaces, increased pleomorphism, mitotic activity, and focal necrosis. The cells in this group had eosinophilic clear cytoplasm, angular nuclei, and coarse chromatin. Perineural and perivascular invasion are common, especially in cribriform and solid patterns.

### Immunohistochemical staining results

#### IHC expression of PHH3 in MEC

The associations of PHH3 levels in MEC patients with clinical parameters, including age, sex, MEC tumor site, and TNM stage, are shown in Table 1. There was a significant difference (*p* = 0.016) in PHH3 expression between the various TNM clinical stages in MEC patients.

**Table 1:** PHH3 IHC expression in MEC in relation to age, gender, tumor, and TNM stages in MEC and in the studied TNM stages of MEC.

	PHH3 final score	Total			Pearson X^2^
		Score 1	Score 2	Score 3	
MEC age groups					
≤ 61 years					
Count	0	5	5	10	0.108
% within MEC age groups	0%	50%	50%	100%	
> 61 years					
Count	2	1	3	6	
% within MEC age groups	33.3%	16.7%	50%	100%	
Total					
Count		6	8	16	
% within MEC age groups	2	37.5%	50%	100%	
MEC gender groups					
Male					0.158
Count	12.5%	0	3	3	
% within MEC gender groups	0%	0%	100%	100%	
Female					
Count	2	6	5	13	
% within MEC gender groups	15.4%	46.2%	38.5%	100%	
Total					
Count	2	6	8	16	
% within MEC gender groups	12.5%	37.5%	50%	100%	
MEC site groups					
Major SG					0.319
Count	2	6	6	14	
% within MEC site groups	14.3%	42.9%	42.9%	100%	
Minor SG					
Count	0	0	2	2	
% within MEC site groups	0%	0%	100%	100%	
Total					
Count	2	6	8	16	
% within MEC site groups	12.5%	37.5%	50%	100%	
MEC TNM stages					
Stage I					0.016
Count	2	1	0	3	
% within MEC TNM stages	66.7%	33.3%	0.0%	100.0%	
Stage III					
Count	0	5	6	11	
% within MEC TNM stages	0.0%	45.5%	54.5%	100%	
Stage IV					
Count	0	0	2	2	
% within MEC TNM stages	0%	0%	100%	100%	
Total					
Count		6	8	16	
% within MEC TNM stages	12.5%	37.5%	50%	100%	

Significance is when *p* value is ≤ 0.05.

**Fig. 2:**
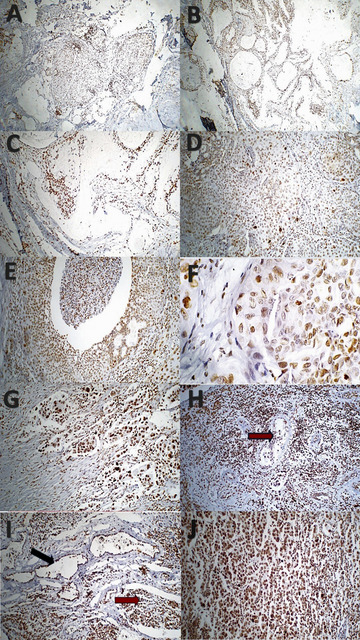
Photomicrograph shows PHH3 immunoreactivity in mucoepidermoid carcinoma (MEC) across tumor grades. Firstly, for score 1 low-grade MECs (**A**) positivity in solid nests (ABC-DAB X 40); (**B**) positivity around multiple macrocystic spaces (ABC-DAB X 40); (**C**) positivity in intermediate cells; mucous cells are largely negative (ABC-DAB X 100). Secondly, for score 2 intermediate-grade MECs (**D**) variable staining intensity, stronger in pleomorphic nuclei (ABC- DAB X 200), (**E**) positivity in cells adjacent to necrosis (ABC-DAB X 200). (**F**) higher magnification showing malignant cells with positive staining (ABC-DAB X 400), thirdly, score 3 High-grade MECs (**G**) marked nuclear immunoreactivity in pleomorphic and multinucleated cells (ABC-DAB × 200); (**H**) intravascular infiltration with strong staining (red arrow) (ABC-DAB × 200); (**I**) positive staining in cells surrounding macrocystic (black arrow) and microcystic (red arrow) spaces (ABC-DAB × 200); (**J**) positivity in loose sheets of malignant cells (ABC-DAB × 200).

In terms of PHH3 IHC expression in relation to the histological grade of MEC, among the high-grade MECs (10 cases), the expression of PHH3 was predominantly score 3. Two of the three patients with low-grade MEC (66.7%) had a PHH3 expression score of 1. All intermediate-grade MECs (3 cases) presented a score of 2 for PHH3 expression. A statistically significant difference in PHH3 immuno-expression was found concerning the MEC histologic grade via Pearson’s chi-square test (*p* = 0.002). PHH3 expression and MEC histologic grade were significantly positively correlated (Pearson’s *R* = 0.840, *p* < 0.000), as shown in Table 2 and Fig. 2.

**Table 2:** Difference of PHH3 IHC and XIAP expression among low, intermediate, and high-grade MECs.

MEC histologic grade	PHH3 final score			Total	Pearson X^2^/*P* value
	Score 1	Score 2	Score 3		
Low grade	2(66.7%)	1(33.3%)	0	3(10%)	0.002
Intermediate grade	0	3(100%)	0	3(10%)	
High grade	0	2(20%)	8(80%)	10 (33.3%)	
Total	2(12.5%)	6(37.5%)	8(50%)	16(53.3%)	

MEC histologic grade (n = 16)	XIAP final score			Total	Pearson X^2^/*P* value
	Weak	Moderate	Strong		
Low-grade MEC (n = 3)	2(66.7%)	1(33.3%)	0	18.75%	0.008
Intermediate grade MEC (n = 3)	0	3 (100%)	0	18.75%	
High-grade MEC (n = 10)	0	4(40%)	6(60%)	62.5%	
Total (n = 16)	2(12.5%)	8(50%)	6(37.5%)	100%	

Significance is when *P* value is ≤ 0.05.

#### XIAP IHC expression in MECs

Half of the MEC group exhibited moderate XIAP expression, whereas strong immunoreaction was observed in 37.5% (6/16) of the MEC group. Weak expression was detected in only two patients (2/16, 12.5%). The relationship of the XIAP in MEC with clinical parameters, including age, sex, the site of the tumor in MEC and TNM stage, was published previously^26^. There was a statistically insignificant difference between XIAP expression in MECs and age, sex, and anatomical site. However, there was a significant difference in XIAP expression across the various TNM clinical stages in the MEC group. (*p* = 0.036).

Two patients (66.7%) with low-grade MEC presented weak XIAP expression compared with the various histologic grades of MEC. Most of the high-grade genes (60%) presented strong expression. Moderate XIAP expression was observed in all three intermediate-grade MECs. Pearson’s chi-square test revealed a substantial statistically significant difference in XIAP IHC expression among the different MEC histologic grades (*p* = 0.008). Furthermore, Table 2 shows a strong positive correlation between XIAP expression and MEC histologic grade (Pearson’s R = 0.725).

**Fig. 3:**
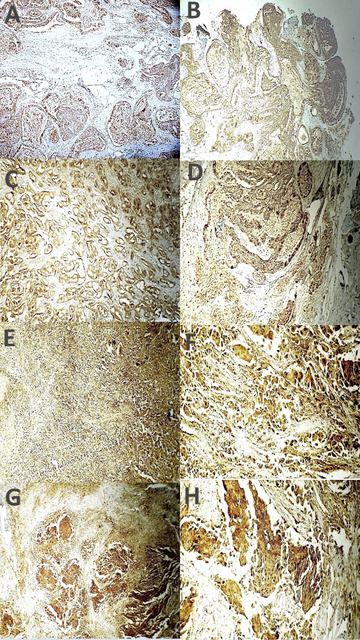
Photomicrograph shows XIAP expression in mucoepidermoid carcinoma (MEC) across tumor grades. Firstly, low-grade MECs (**A**) Moderate expression (ABC DAB × 100); (**B**) Weak expression (ABC DAB × 40); (**C**) Moderate expression (ABC DAB × 200). Secondly, for high-grade MECs (**D**) Strong expression in scattered malignant glandular epithelial cells (ABC DAB × 40); (**E**) Strong expression in small glandular epithelial foci of epidermoid cells (ABC DAB × 200); (**F**) Strong expression in solid follicles with abundant sclerosis (ABC DAB × 40); (**G**) Strong membranous and cytoplasmic expression (ABC DAB × 200); (**H**) Strong membranous and cytoplasmic expression (ABC DAB × 200).

One-way ANOVA with post hoc tests for multiple comparisons revealed strong statistically significant differences in XIAP expression across the three histologic grades of MEC patients concerning the expression of the protein across the various MEC histologic grades. XIAP expressions in high-grade MECs were significantly greater than those in low-grade MECs (*p* = 0.002). Additionally, there was a statistically insignificant difference in XIAP expression between low- and intermediate-grade carcinomas (*p* = 0.117), as shown in Table 3 and Fig. 3.

**Table 3:** Multiple comparisons between the MEC histologic grades concerning XIAP expression using One- Way ANOVA post-hoc test.

(I) MEC histologic grade	(J) MEC histologic grade	Mean difference (I–J)	Std. error	Sig.	95% confidence interval	
					Lower bound	Upper bound
Low grade	Intermediate grade	− 0.667	0.397	0.117	− 1.52	0.19
	High grade	− 1.267*	0.320	0.002	− 1.96	− 0.58
Intermediate grade	Low grade	0.667	0.397	0.117	− 0.19	1.52
	High grade	− 0.600	0.320	0.083	− 1.29	0.09
High grade	Low grade	1.267*	0.320	0.002	0.58	1.96
	Intermediate grade	0.600	0.320	0.083	− 0.09	1.29

*Significance is when *P* value is ≤ 0.05.

#### IHC expression of PHH3 in AdCCs

PHH3 expression in AdCC patients in relation to different clinical parameters, including age, sex, anatomical site, and histological grade, is shown in Table 4. There was a statistically insignificant difference in PHH3 expression between the two age groups and between the two sexes.

**Table 4:** PHH3 in AdCC in relation to different clinical parameters including age, gender, the site of the tumor in AdCC and AdCC stages.

	PHH3 final score		Total	Pearson X^2^
	Score 2	Score 3		
ADCC age groups				
≤ 57 years				
Count	5	4	9	0.387
% within ADCC age groups	55.6%	44.4%	100%	
> 57 years				
Count	0	5	5	
% within ADCC age groups	0.0%	100%	100%	
Total				
Count	5	9	14	
% within ADCC age groups	35.7%	64.3%	100%	
ADCC gender groups				
Male				
Count	3	4	7	0.577
% within ADCC gender groups	42.9%	57.1%	100%	
Female				
Count	2	5	7	
% within ADCC gender groups	28.6%	71.4%	100%	
Total				
Count	5	9	14	
% within ADCC gender groups	35.7%	64.3%	100%	
ADCC site groups				
Major SG				
Count	1	7	8	0.036
% within ADCC site groups	12.5%	87.5%	100%	
Minor SG				
Count	4	2	6	
% within ADCC site groups	66.7%	33.3%	100%	
Total				
Count	5	9	14	
% within ADCC site groups	35.7%	64.3%	100%	
ADCC TNM stages				
Stage II				
Count	1	0	1	0.233
% within ADCC TNM stages	100.0%	0.0%	100.0%	
Stage III				
Count	4	7	11	
% within ADCC TNM stages	36.4%	63.6%	100.0%	
Stage IV				
Count	0	2	2	
% within ADCC TNM stages	0%	100%	100%	
Total				
Count	5	9	14	
% within ADCC TNM stages	35.7%	64.3%	100%	

**Fig. 4:**
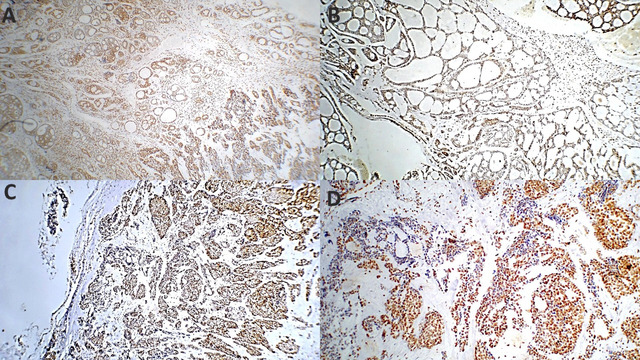
Photomicrograph shows PHH3 immunoreactivity in adenoid cystic carcinoma (AdCC) across histological patterns. (**A**) Tubular patterns score 1 immunoreactivity (ABC-DAB X 40); (**B**) Cribriform pattern score 2 immunoreactivity (ABC-DAB X 200); Solid pattern score 3 immunoreactivity (**C**) (ABC-DAB X 40); (**D**) (ABC-DAB X 200).

With respect to PHH3 IHC expression in relation to AdCC histologic grade, all grade 3 AdCCs (8/14 cases) presented a score of 3 for PHH3 expression, whereas four out of five (80%) grade 2 AdCCs presented a score of 2. The single case representing grade 1 AdCC had a score of 2 for PHH3 expression. A substantial difference in PHH3 immunoexpression was found in relation to the AdCC histologic grade, as indicated by Pearson’s chi-square test (*p* = 0.005). A strong positive correlation between PHH3 expression and AdCC histologic grade was detected (Pearson’s R = 0.832, *p* < 0.001), as shown in Table 5 and Fig. 4.

**Table 5:** Difference of PHH3 IHC and XIAP expression to the different histologic grades of AdCC.

ADCC histologic grade	PHH3 final score		Total	Pearson X^2^/*P* value	
	Score 2	Score 3			
Grade 1 (tubular pattern)	1/1(100%)	0/1	1/14(7.1%)	0.005	
Grade 2 (cribriform pattern)	4/5(80%)	1/5(20%)	5/14(35.7%)		
Grade 3 (solid component > 30%	0/8	8/8(100%)	8/14(57.1%)		
Total	5/14(35.7%)	9/14(64.3%)	14(100%)		

AdCC histologic grade	XIAP final score			Total	Pearson X^2^/*P* value
	Weak	Moderate	Strong		
Grade 1 (tubular pattern)	1(100%)	0	0	1(7.14%)	0.000
Grade 2 (cribriform pattern)	0	5(100%)	0	5(35.7%)	
Grade 3 (≥ 30% solid pattern)	0	3(37.5%)	5(62.5%)	8(57.1%)	
Total	1(7.1%)	8(57.1%)	5(35.7%)	14	

#### IHC expression of XIAP in AdCC

The XIAPs in AdCCs in relation to clinical parameters, including age, sex, anatomical site, and grading, are shown in Table 6. There were statistically significant differences in XIAP immunoreactivity when comparing the various anatomical areas of tumor presentation.

**Table 6:** XIAP IHC expression in AdCC in relation to in relation to different clinical parameters including age, gender, the site of the tumor in AdCC and AdCC stages.

	XIAP final score			Total	Pearson X^2^
	Weak	Moderate	Strong		
AdCC age groups					
≤ 57 years					
Count	1	5	3	9	0.738
% within ADCC age groups	11.1%	55.6%	33.3%	100.0%	
> 57 years					
Count	0	3	2	5	
% within ADCC age groups	0.0%	60.0%	40.0%	100.0%	
Total					
Count	1	8	5	14	
% within ADCC age groups	7.1%	57.1%	35.7%	100.0%	
ADCC gender groups					
Male					
Count	0	5	2	7	0.427
% within ADCC gender groups	0.0%	71.4%	28.6%	100.0%	
Female					
Count	1	3	3	7	
% within ADCC gender groups	14.3%	42.9%	42.9%	100.0%	
Total					
Count	1	8	5	14	
% within ADCC gender groups	7.1%	57.1%	35.7%	100.0%	
ADCC site groups					
Major SG					
Count	1	2	5	8	0.019
% within ADCC site groups	12.5%	25.0%	62.5%	100.0%	
Minor SG					
Count	0	6	0	6	
% within ADCC site groups	0.0%	100.0%	0.0%	100.0%	
Total					
Count	1	8	5	14	
% within ADCC site groups	7.1%	57.1%	35.7%	100.0%	
MEC TNM stages					
Stage II					
Count	1	0	0	1	0.007
% within ADCC TNM stages	100.0%	0.0%	0.0%	100%	
Stage III					
Count	0	7	4	11	
% within ADCC TNM stages	0.0%	63.6%	36.4%	100%	
Stage IV					
Count	0	1	1	2	
% within ADCC TNM stages	0.0%	50.0%	50.0%	100%	
Total					
Count	1	8	5	14	
% within ADCC TNM stages	7.1%	57.1%	35.7%	100%	

**Fig. 5:**
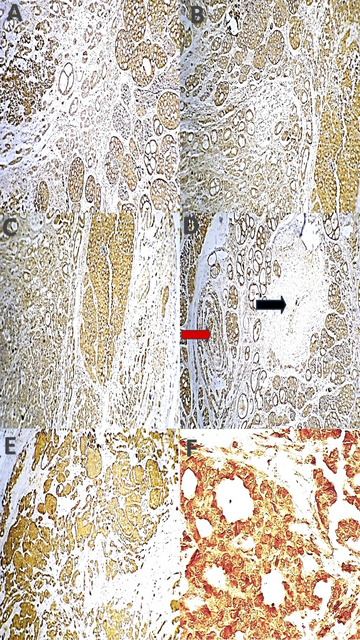
Photomicrograph shows XIAP expression in adenoid cystic carcinoma (AdCC) across histological patterns. Firstly, Tubular pattern (**A**) Weak expression with occasional strong reactivity; ductal cells are reactive while myoepithelial cells show occasional positivity (ABC-DAB X 200); (**B**) Moderate expression with occasional strong reactivity in tubular and cribriform areas (ABC-DAB X 200); Secondly, cribriform pattern (**C**) Moderate expression with occasional strong reactivity (ABC-DAB X 200); (**D**) Moderate expression with intravascular (black arrow) and perineural invasion (red arrow) (ABC-DAB X 200); Thirdly, solid pattern (**E**) Moderate expression with occasional strong reactivity (ABC-DAB X 200); (**F**) Strong cytoplasmic and membranous expression (ABC-DAB X 400).

With respect to XIAP expression with respect to the AdCC histologic grade, all the sections associated with grade 1 AdCCs presented weak XIAP expression, whereas all grade 2 AdCCs presented moderate immunoreactivity. In more than half (62.5%) of the Grade 3 AdCCs, the immunoreactivity for XIAP was strong. There was a significant difference in the expression of XIAP via IHC between the various histologic grades of ADCC patients. Moreover, there was a strong positive correlation between XIAP expression and AdCC histologic grade, as shown in Table 5 and Fig. 5.

### Comparison of PHH3 and XIAP immunohistochemical expression between AdCC and MEC patients

Upon correlating PHH3 and XIAP IHC expression in MEC patients with their expression in AdCC patients via the Pearson chi-square test, a statistically insignificant difference was detected between the two histologic types of SGC patients (Table 7). There was a strong positive correlation between XIAP and PHH3 expression (Pearson’s R = 0.749 and 0.840; relatively, the IHC expression of PHH3 and XIAP was similar across the different MEC histologic grades. There was a strong positive correlation between XIAP and PHH3 expressions (Pearson’s R = 0.774 and 0.832). Similarly, the IHC expression of PHH3 and XIAP was similar across the different AdCC histologic grades.

**Table 7:** PHH3 and XIAP IHC expression in MEC in relation to the tumor histologic type.

Tumor histologic type	PHH3 final score			Total	Pearson X^2^/*P* value
	Score 1	Score 2	Score 3		
Mucoepidermoid carcinoma	2(12.5%)	6(37.5%)	8(50.0%	16(53.3%)	0.363
Adenoid cystic carcinoma	0	5(35.7%)	9(64.3%)	14(46.7%)	
Total	2(6.7%)	11(36.7%)	17(56.7%)	30(100%)	

MEC histologic grade (n = 16)	XIAP final score			Total	Pearson X^2^/*P* value
	Weak	Moderate	Strong		
MEC	2(12.5%)	8(50%)	6(37.5%)	16(53.3%)	0.189
AdCC	1(7.1%)	8(57.1%)	5(35.7%)	14(46.7%)	
Total	6(20%)	13(43.3%)	11(36.7%)	30(100%)	

## Discussion

MECs and AdCCs are the most common types of malignant salivary gland tumors. Both show wide morphological variation and behave differently across cases^1^. Malignant salivary gland tumors exhibit wide variation in their histopathological features, which may contribute to differences in clinical behavior and prognosis. The behavior of salivary gland tumors such as MECs and AdCCs, which are the two most common MSGTs, still has little predictability because of the lack of well-delineated prognostic factors; therefore, trials that provide clues and hints about the recognition of proteins that control biological processes such as mitosis and apoptosis are mandatory for more precise evaluation and, consequently, better therapeutic strategies. This study used immunohistochemistry to examine PHH3 and XIAP expression in MECs and AdCCs. The goal of this study was to evaluate whether the expression levels of these markers reflect the biological activity driving tumor behavior.

For the MEC group, the mean age (61.4 years) was in line with that reported by many other investigators^27,28^ and was approximately five to ten years older than the mean age reported by others^29,30^. Despite the small number of patients in the currently studied AdCC group, the mean age (56.9 years) was approximate and comparable to the previously reported mean age in larger series^31,32^. Concerning the anatomical site of tumor involvement, the majority of the currently studied SGCs arise from MSGs, particularly from the parotid gland. This finding agreed with those of prior studies^33^. Researchers have reported that the aggressive clinical behavior of salivary carcinoma is significantly correlated with parotid gland involvement^34,35^. This finding may explain why the majority of the studied MECs and AdCCs were in the parotid gland and were reported to have nodal involvement, assigning them to advanced clinical stages (III and IV). Moreover, Grade I AdCC and low-grade MEC cases were reported for TNM stages I & II. Similar results were documented in previous studies^36,37^. In terms of TNM stage, MEC patients with advanced clinical stage (III and IV) disease presented significantly greater PHH3 immuno-expression. Similar findings have been reported by other researchers with respect to ovarian tumors^38^. In contrast, other reports have shown an insignificant correlation between disease stage and PHH3 expression in neuroendocrine tumors of the pancreas^39^ and breast cancer^12^. Notably, the relationships among PHH3 expression, tumor behavior, and disease stage might vary depending on the specific type of tumor and individual patient characteristics^11^.

In terms of the association with histological grade, the present study revealed a significant strong positive correlation between the PHH3 score and the histologic grade of the MEC patients. Many studies of other tumors are consistent with our findings concerning this point, reporting that the proliferative activity determined by PHH3 is positively correlated with tumor grade in prostate cancer^40^, ovarian tumors^38^, and breast adenocarcinomas^41^. The consistent correlation was confidently conducive to employing PHH3 scoring as a replacement for grading breast cancer^42^. In contrast, studies conducted on pancreatic neuroendocrine tumors have shown statistically insignificant differences in PHH3 expression related to histologic grade^39^.

The age and sex of the working MEC patients were not significantly associated with high PHH3 expression. Similarly, Tracht et al.^39^ reported no significant correlation between PHH3 immunoexpression and patient age in patients with pancreatic neuroendocrine tumors. Moreover, Villani et al.^43^ reported that PHH3 was not significantly correlated with age or sex in patients with pancreatic neuroendocrine tumors. In contrast to our findings, other investigators reported a statistically significant correlation between high PHH3 immunoexpression and advanced age in patients with breast cancer. In contrast, Skaland et al.^42^ reported that decreased PHH3 expression was associated with advanced age in patients with breast cancer. With respect to the anatomical site of the worked MEC patients, there was a statistically insignificant difference in the scores of PHH3 IH expression among the anatomical sites. With respect to the expression of XIAP, the TNM clinical stage of the worked MECs revealed highly statistically significant differences, where higher XIAP scores were recorded in the MEC patients reported with advanced clinical stages. This association was in line with that reported by Ning et al., who reported a significant correlation between high XIAP expression and higher clinical stage in patients with malignant salivary gland tumors^44^. Moreover, high XIAP expression was significantly correlated with advanced clinical stage in hepatocellular and pancreatic carcinoma^45^.

In the present study, high XIAP expression was also significantly associated with higher histologic grades in MEC patients, with a strong positive correlation. Similarly, high XIAP expression was found in patients with higher histologic grades of pancreatic cancer^46^ and breast ductal carcinomas^47^. However, some studies on breast cancer reported the opposite results^24^ and^48^. High XIAP expression is related to the molecular changes that occur during tumor progression, leading to higher histologic grades since it plays a significant role as an antiapoptotic protein that inhibits cell death. Thus, overexpression may aid cancer cell proliferation and survival, which could result in more aggressive tumor characteristics.

This might provide an answer to the question of why higher histologic grades in MEC patients are often associated with more aggressive tumor behavior and increased tumor progression.

While XIAP expression was significantly higher in high-grade MECs than in low-grade MECs, there was no significant difference in XIAP expression between high- and intermediate-grade MECs. Additionally, there was a statistically insignificant difference in XIAP expression between low- and intermediate-grade carcinomas. These findings suggest that the malignant potential of intermediate-grade MEC is comparable to that of low- or high-grade MEC. Thus, the current results stand in line with the earlier classification of^49^, who advocated for the exclusive classification of MEC into low and high grades.

With respect to the variation in XIAP immunoexpression in the evaluated clinical parameters of the worked MEC group, statistically insignificant differences were observed in the XIAP score with respect to patient age or sex. A similar insignificant correlation was reached in reported patients with pancreatic^46^ and breast cancer^48^. These conflicting findings could be attributed to differences in sample size and selection criteria. Studies with smaller sample sizes may not accurately represent the overall population, leading to variations in the observed results. Additionally, variations in patient demographics, such as age and sex distributions, can also contribute to conflicting findings. Moreover, differences in the methodologies used to assess XIAP expression can also contribute to conflicting results. Variations in the techniques, antibodies, and scoring systems employed by different research groups can lead to discrepancies in the reported XIAP expression levels. In terms of the anatomical site of MEC, the present research revealed no statistically significant difference in the XIAP expression scores between the major and minor salivary gland groups. Similarly, Ning et al.^44^ reported that XIAP expression did not correlate with the primary tumor site in malignant salivary gland tumors. The differences in the study design and sample characteristics between the current study and the study by Ning et al.^44^ could contribute to the conflicting findings. The differences in the study design and sample characteristics between the current study and the study by Ning et al.^44^ could contribute to the conflicting findings. A larger sample size of Ning et al.’s study (95 patients with malignant salivary gland tumors, including 50 AdCCs and 45 MECs) compared with our cohort of 30 patients increases statistical power and may reveal associations not evident in smaller studies.

Regarding the anatomical site of the worked AdCC cases, there was a highly statistically significant association between PHH3 expression in major SGs and that in minor salivary glands. Additionally, concerning the histological grade of AdCC, the present study demonstrated a significantly strong positive correlation between the PHH3 score and the histological grade. Many studies of other tumors are consistent with our findings concerning this point, reporting that the proliferative activity determined by PHH3 is positively correlated with tumor grade in prostate cancer^40^, ovarian^38^, and breast adenocarcinomas^41^. This consistent correlation is conducive to the use of PHH3 for grading tumors such as breast cancer^42^. In contrast, studies conducted on pancreatic neuroendocrine tumors have shown no statistically significant difference in PHH3 expression according to histologic grade^39^. Among the worked AdCC group, neither age nor sex were significantly associated with the PHH3 expression score. Similarly, no significant correlation was found between PHH3 immunoexpression and patient age in patients with pancreatic neuroendocrine tumors^39^. Moreover, Villani et al. reported that PHH3 was not significantly correlated with age or sex in patients with pancreatic neuroendocrine tumors^43^. Skaland et al.^42^ reported that decreased PHH3 expression was associated with advanced age in patients with breast cancer. Compared with patients with earlier TNM stages, those with advanced clinical stages (III and IV) presented insignificantly high PHH3 immunoexpression. Similar findings in other reports revealed an insignificant correlation between disease stage and PHH3 expression in neuroendocrine tumors of the pancreas^39^ and in breast cancer^12^. In contrast, a significant direct association between PHH3 expression and clinical stage has been reported by other researchers in breast cancer and ovarian tumors^38^. Notably, the relationships among PHH3 expression, tumor behavior, and disease stage can vary depending on the specific type of tumor and individual patient characteristics.

Regarding the anatomical site of the worked AdCC group, the present findings revealed a significantly higher score of XIAP expression in AdCC cases that arose from the major salivary glands than in those that arose from the minor salivary glands. Given that the parotid gland is the most common site of AdCC (8/14, 57%) and that proximity to facial nerve perineural invasion is a common result of AdCC, the overexpression of XIAP in the parotid gland in AdCC may be due to the “low-resistance” hypothesis, which views the perineural space as a low-resistance channel through which neoplastic cells can easily spread along the nerve^6^.

The distribution of the expression scores of the XIAP among the TNM clinical stages of the worked AdCC group was significantly different, where high XIAP expression was recorded in the advanced clinical stages of AdCC patients. This association agreed with the findings of Ning et al. who reported a significant correlation between high XIAP expression and higher clinical stage in MSGTs^44^. Moreover, high XIAP expression was significantly correlated with advanced clinical stage in hepatocellular and pancreatic carcinoma^46,47^. In contrast, Schnoell et al.^50^ reported the absence of any significant correlations between different clinicopathological factors and the expression of XIAP in AdCC patients.

In the present study, strong XIAP expression was significantly associated with higher histologic grades in the AdCC group, with a strong positive correlation (*p* < 0.001, *R* = 0.774). Similarly, high XIAP expression was noted in patients with higher histologic grades of pancreatic cancer^46^ and breast ductal carcinomas^47^. In contrast, opposite findings have been reported in other studies on breast cancer^26,49^. The molecular alterations that take place during tumor progression and result in higher histologic grades could be connected to elevated XIAP expression. The overexpression of XIAP, an antiapoptotic protein that prevents cell death, may help cancer cells survive and proliferate, which could result in more aggressive tumor features^17^. With respect to the relationship of XIAP immunoexpression with patient age and sex in AdCC patients, we detected statistically insignificant differences in XIAP immunoexpression according to patient age and sex, similar to the findings of^46^ in pancreatic cancer patients. Similarly, Zhang et al.^48^ reported no correlation between XIAP immunoexpression and patient age in patients with breast cancer. In contrast to our findings, Schnoell et al.^50^ reported significantly higher XIAP expression in women than in men with AdCC.

The significant positive correlation observed between PHH3 and XIAP expression in our study highlights the potential complementary roles of these biomarkers in salivary gland tumor progression. PHH3, as a mitotic marker, identifies cells actively engaged in chromosomal segregation, thus reflecting the proliferative fraction of the tumor. XIAP, in contrast, acts downstream in the apoptotic cascade by directly inhibiting effector caspases, thereby prolonging tumor cell survival. The simultaneous upregulation of these markers in higher histologic grades of both MEC and AdCC suggests that enhanced proliferation coupled with suppressed apoptosis may act in concert to drive tumor aggressiveness. This proliferative–antiapoptotic axis could partly explain the association of high PHH3 and XIAP levels with adverse clinicopathological parameters and supports the hypothesis that evaluating both markers together may provide a more comprehensive assessment of tumor biology than assessing either in isolation.

This study has some limitations that should be acknowledged. First, the sample size was relatively small, with only a single low-grade AdCC case, which reduces statistical power and limits generalizability, but it was related to the rarity of the condition and feasibility constraints. Second, the retrospective, single-institution design may not fully represent the broader spectrum of salivary gland malignancies and introduces inherent constraints. Third, the use of archived tissue blocks carries the possibility of selection bias, as only available and suitable samples were included. Finally, the lack of outcome data, such as patient survival or recurrence rates, prevents definitive assessment of the prognostic significance of PHH3 and XIAP. Nevertheless, the consistency of our associations across both tumor types and alignment with previously reported trends in other malignancies strengthen the validity of our observations and provide a solid basis for larger, multi-center prospective studies with comprehensive clinical follow-up to validate and expand upon the current findings.

In conclusion, this study shows that PHH3 and XIAP expression levels are associated with higher histologic grades in MEC and AdCC. PHH3 expression was also associated with advanced clinical stage in MEC. These findings suggest a potential link between marker expression and tumor aggressiveness. However, due to the small sample size and retrospective design, the results should be interpreted with caution. Larger, prospective studies are needed to validate the clinical and prognostic significance of PHH3 and XIAP in salivary gland malignancies.

## Data Availability

All data generated or analyzed during this study are included in this published article.

## Abbreviations

AdCC: Adenoid cystic carcinoma
MEC: Mucoepidermoid carcinoma
PHH3: Phosphohormone H3
XIAP: X-linked inhibitor of apoptosis protein
MSGTs: Malignant salivary gland tumors
IHC: Immunohistochemistry
IAP: Inhibitor of apoptosis
ABC: Avidin–biotin complex
SPSS: Statistical package for social science
SD: Standard deviation
